# Effectiveness and cost-effectiveness of the modified Pilates method versus aerobic exercise in the treatment of patients with fibromyalgia: protocol for a randomized controlled trial

**DOI:** 10.1186/s41927-018-0051-6

**Published:** 2019-01-18

**Authors:** Katherinne Ferro Moura Franco, Yuri Rafael dos Santos Franco, Evany Maira Espírito Santo Salvador, Bruna Cristina Brajon do Nascimento, Gisela Cristiane Miyamoto, Cristina Maria Nunes Cabral

**Affiliations:** 1 Master’s and Doctoral Program in Physical Therapy, Universidade Cidade de São Paulo, Rua Cesário Galeno 475, São Paulo SP, CEP: 03071-000 Brazil; 2 Physical Therapy Department, Universidade Cidade de São Paulo, Rua Cesário Galeno 475, São Paulo SP, CEP: 03071-000 Brazil

**Keywords:** Pilates method, Aerobic exercise, Economic evaluation

## Abstract

**Background:**

Fibromyalgia is characterized by chronic generalized pain, fatigue, sleep disorders and other symptoms. Physical exercise is recommended as the first choice of non-pharmacological therapy. Thus, the aim of this study is to evaluate the effectiveness and cost-effectiveness of modified Pilates exercises compared to aerobic exercises in the treatment of patients with fibromyalgia.

**Methods:**

In this randomized controlled trial with blinded assessor, 98 patients who meet the fibromyalgia classification criteria of the American College of Rheumatology 2010, aged between 20 and 75 years, and with pain intensity greater than or equal to 3 points in the Pain Numerical Rating Scale, will be randomly divided into Aerobic Group (aerobic exercises on treadmills or stationary bikes) and Pilates Group (modified Pilates exercises), and treated twice a week for eight weeks on the Center for Excellence in Clinical Research in Physical Therapy at Universidade Cidade de São Paulo, Brazil. The following outcomes will be evaluated by a blinded assessor at baseline, eight weeks, six months, and 12 months after randomization: impact of fibromyalgia assessed by the Fibromyalgia Impact Questionnaire, pain intensity by the Pain Numerical Rating Scale, kinesiophobia by the Tampa Scale of Kinesiophobia, specific disability by the Patient-Specific Functional Scale, functional capacity by the 6-min Walk Test, quality of sleep by the Pittsburgh Sleep Quality Index, and health-related quality of life by EQ-5D-3L and SF-6D questionnaires.

**Discussion:**

It is expected that the Pilates exercises will be more effective than aerobic exercises in improving clinical outcomes and that this improvement will be maintained over the medium to long term. This study aims to clarify whether the Pilates method can be incorporated into the clinical practice of physical therapists treating patients with fibromyalgia. The study will also provide information on which exercise will be most cost-effective, information that can be used by insurers and public health systems.

**Trial registration:**

This study was prospectively registered at the Clinical Trials Registry (NCT03050606) in February 2017.

**Electronic supplementary material:**

The online version of this article (10.1186/s41927-018-0051-6) contains supplementary material, which is available to authorized users.

## Background

Fibromyalgia is a rheumatologic disease characterized by chronic generalized pain, hyperalgesia, and allodynia [1]. It is also characterized by the presence of symptoms such as fatigue, sleep disorders, morning stiffness, headache, and paresthesia [1]. It can be classified clinically, following the diagnostic criteria of the American College of Rheumatology 2010 [2]: 1) Widespread Pain Index (WPI), calculated by adding up the number of areas in which the patient reported pain in the last week; and 2) Severity of Symptoms (SS), calculated by adding up the severity of fatigue, waking unrefreshed, cognitive symptoms and somatic symptoms [2]. To be classified as having fibromyalgia, a patient must present the following three clinical conditions: WPI ≥ 7 and SS ≥ 5 or WPI between 3 and 6 and SS ≥ 9 points; presence of symptoms at the same intensity for at least three months; and no disease/condition that could be the cause of the pain [2]. One of the main pathophysiological mechanisms of fibromyalgia is central sensitization, defined as increased responsiveness of nociceptive neurons in the central nervous system to their normal or subthreshold afferent input [3]. In this case, pain can arise spontaneously or be elicited by a normally innocuous stimuli (allodynia), is exaggerated and prolonged in response to a noxious stimuli (hyperalgesia), and spreads beyond the site of injury (secondary hyperalgesia) [4].

The average global prevalence of fibromyalgia is 2.7%, with a higher incidence in women (3:1) [5, 6]. In Brazil, this prevalence is 4.4% in the population aged 35–60 years [7] and 5.5% in people over 65 years of age [8]. Economic impact studies show that patients with fibromyalgia are significant consumers of health services and generate significant costs related to loss of productivity [9]. The average annual cost of fibromyalgia is estimated at approximately 6000 Canadian dollars [10], approximately 2,827,000 Yen of indirect cost and 1,943,000 Yen of direct cost in Japan [11], and this cost is even higher in European patients, approximately 8000 to 13,000 Euros per year [12, 13].

The current recommendation for the treatment of fibromyalgia includes non-pharmacological and self-management strategies combined with pharmacological therapies [14]. Exercise is the first choice of treatment, with stronger evidence for supervised aerobic exercise. However, there is also evidence recommending muscle strengthening and stretching exercises [5, 14, 15]. Some studies [15–17] demonstrate that aerobic exercise improves fibromyalgia symptoms when compared to control groups or other interventions (such as education and cognitive-behavioral therapy). However, the size of these effects is small to moderate [16, 17]. Another type of exercise that can be a good option for the treatment of these patients is the modified Pilates method, as it has exercises to mobilize, stretch, and strengthen muscles. The modified Pilates is currently the most widely used approach by physical therapists, is tailored to the practitioner, divided into levels of progression, and emphasizes the normal curvatures of the spine during the exercises [18]. Pilates exercises are often indicated for the treatment of low back pain [19, 20], but there are only four pilot studies on the effects of the Pilates method in patients with fibromyalgia [21–24]. These studies demonstrate that Pilates may be better than short-term exercise at home [21] and connective tissue massage for pain-pressure threshold and anxiety [22], but similar to yoga exercises [24] and a control group without intervention [23] in patients with fibromyalgia.

A systematic review [25] showed that there is substantial evidence that various modalities of exercise improve pain and function in patients with fibromyalgia but also detected inconclusive evidence for new modalities such as Pilates, yoga and tai chi because of the lack of studies and methodological flaws of the existing studies. This demonstrates the need for studies with adequate comparator groups and samples to verify the actual effectiveness of modified Pilates exercises in this disease. In addition, fibromyalgia generates high costs in health, but there is little information about the cost-effectiveness of the treatments indicated for its control, and it is extremely important to identify the most effective and less costly treatments. Thus, the aim of this study is to evaluate the effectiveness and cost-effectiveness of modified Pilates exercises compared to aerobic exercises in the treatment of patients with fibromyalgia.

## Methods

### Study design

This study is a randomized controlled trial with parallel group and blinded assessor.

### Study settings

**Fig. 1:**
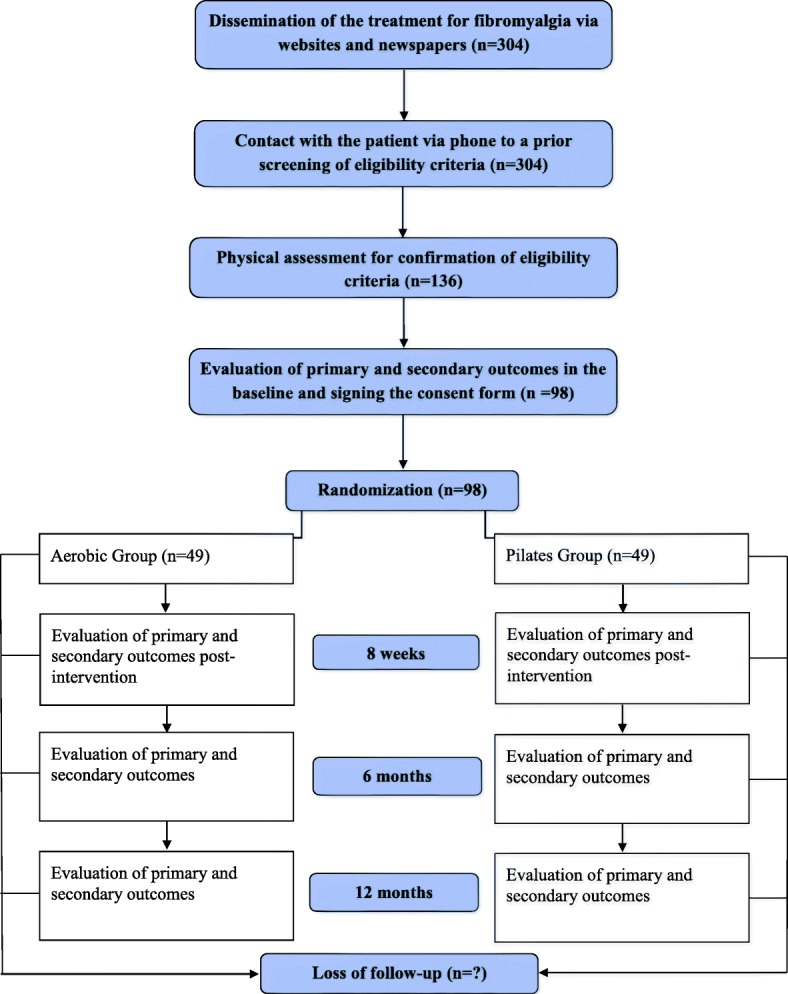
Study design

Participants will be treated at the Center for Excellence in Clinical Research in Physical Therapy at Universidade Cidade de São Paulo in Sao Paulo, SP, Brazil. This project was approved by the Research Ethics Committee of Universidade Cidade de São Paulo (CAAE: 51328215.1.0000.0064). It was also prospectively registered on Clinical Trials Registry (NCT03050606) and received funding from the Sao Paulo Research Foundation - FAPESP (process number 2016/12962–0 and 2015/21590–6). If there is a need to change the protocol, the changes will be immediately reported to the Research Ethics Committee and carried out at the registration site before the first patient is admitted to the study. The flow of participants through the study will follow the recommendations of the Consolidated Standards of Reporting Trials Statement and is outlined in Fig. 1.

### Sample size calculation

The study was designed to detect a clinically significant 8 points (14%) difference in the impact of fibromyalgia assessed by the Fibromyalgia Impact Questionnaire eight weeks after randomization (estimated standard deviation = 12), assuming α = 0.05, statistical power of 80%, and loss to follow-up of 15%. This value represents the minimal clinically important difference [26]. The sample size calculation resulted in a sample of 98 participants divided into two groups of 49 each. To reach this number, the study will be promoted in community newspapers and on the internet.

### Eligibility criteria

The study will include patients of both genders who meet the fibromyalgia classification criteria of the American College of Rheumatology 2010 [2], aged between 20 and 75 years, and with pain intensity greater than or equal to 3 points in the Pain Numerical Rating Scale [27]. The exclusion criteria will be contraindications to physical exercise [28], pregnancy, uncontrolled systemic diseases (diabetes mellitus, systemic arterial hypertension, and thyroid dysfunctions), neurological, cardiorespiratory, and musculoskeletal conditions that interfere in the treatment, e.g., paralysis, significant loss of sensitivity (such as diabetic foot), changes in level of consciousness and understanding, advanced joint diseases, injury or severe pain in the lower limbs that limit the performance of aerobic exercises (such as severe arthrosis, hip or knee arthroplasty, severe rheumatoid arthritis, cervicalgia with irradiation to the upper limbs, meniscus injury, plantar fasciitis, partial or total muscle tears, amputations, among others), recent surgery, and inability to understand or speak Portuguese. Prior screening will be conducted by telephone by a previously trained student, with exception of irradiated pain, which will be assessed in a face-to-face assessment. Participants will be asked to not look for other treatment (whether based on exercises or non-pharmacological interventions) while undertaking the study intervention.

### Assessment of clinical outcomes

Before baseline assessment, the participants will give consent and sign the Informed Consent Form. A blinded assessor will confirm the eligibility criteria and the diagnosis of fibromyalgia through the criteria of the American College of Rheumatology 2010 [2]. Next, demographic and anthropometric data will be collected, as well as information on the use of medications (type and quantity) for fibromyalgia and physical therapy or other previous treatment. Finally, the participants will be assessed for the primary and secondary outcomes. The assessments will be performed at baseline, eight weeks, six months, and 12 months after randomization, and in the last three assessments, the patients will be instructed not to provide information about their treatment to the blinded assessor. In the follow-ups assessments, patients will also be asked about the use of medication and physical activity or other treatment for fibromyalgia. The baseline and eight-week assessments will be face-to-face, and the six- and twelve-month assessments will be conducted over the phone to minimize loss to follow-up. In the eight-week assessment patients will also be asked about their satisfaction with the treatment. All of the scales and the questionnaire used in this study have already been translated and adapted to Brazilian Portuguese [29–41].

The primary outcome is the impact of fibromyalgia eight weeks after randomization. The secondary outcomes are: impact of fibromyalgia six and 12 months after randomization; pain intensity, kinesiophobia, sleep quality, health-related quality of life and specific disability eight weeks, six months, and 12 months after randomization; pain intensity at each session, before and after treatment; and functional capacity eight weeks after randomization (Table 1).

**Table 1:** Description of the outcome measures

Measure	Construct	Description
Fibromyalgia Impact Questionnaire (FIQ) [30]	Fibromyalgia impact	10-item questionnaire. Item 1 contains 10 questions related to functionality, rated on a 4-point Likert scale ranging from 0 (always) to 3 (never). In items 2 and 3, the participants mark the number of days that they felt well and the number of days they were unable to work due to fibromyalgia in the last seven days. Items 4 to 10 are composed of numerical scales that rate work difficulty, pain, fatigue, morning tiredness, stiffness, anxiety, and depression. The FIQ score ranges from 0 to 100 points and higher values ​​indicate a greater impact of fibromyalgia on quality of life. In general, the scores of patients with fibromyalgia have an average of 50 points, but in those patients with severe impairment, values ​​exceed 70 points.
Pain Numerical Rating Scale (PNRS) [29]	Pain intensity	11-point numerical scale (0 to 10) that ranges from 0 (no pain) to 10 (pain as bad as it could be). In the baseline and follow-up assessments, the participants will rank their average pain in the last seven days. In assessing pain at each session, the participants will rate their pain before and after treatment.
Tampa Scale of Kinesiophobia (TSK) [31, 32]	Kinesiophobia	17-item questionnaire that addresses the fear that physical activity will cause pain or recurrence of the injury, with answers ranging from 1 (strongly disagree) to 4 points (strongly agree). For the final score, it is necessary to reverse the scores of questions 4, 8, 12, and 16. The total score varies between 17 and 68 points, and the higher the score is, the greater the degree of kinesiophobia.
Patient-Specific Functional Scale (PSFS) [29]	Specific disability	Scale in which participants will identify the three main activities that they feel incapable of doing or have difficulty doing because of fibromyalgia. Next, the participants will rate how able they feel capable of performing the activities identified on an 11-point scale, ranging from 0 (unable to perform activity) to 10 (able to perform the activity at preinjury level). The average of the three activities will be calculated, and the higher the score is, the greater the specific capacity of the patient.
Euroqol 5 dimensions (EQ-5D-3L) [41]	Health-related quality of life	Two-part questionnaire: the first part records the severity of the problem in each of the five dimensions of the questionnaire, and the second part contains a 20-cm vertical visual analogue scale ranging from 0 to 100, where 0 corresponds to the worst imaginable health states and 100 corresponds to the best imaginable health states. Its five dimensions are mobility, self-care, usual activities, pain/discomfort, and anxiety/depression, and each dimension has three levels of severity (no problems, moderate problems, and extreme problems). Health status is labeled with a five-digit number that represents the severity level in each dimension. For example, 11111 represents no problem in any dimension, whereas 33333 represents extreme problems in all five dimensions.
Short-Form 6 dimensions (SF-6D) [33, 34, 39, 40]	Health-related quality of life	Questionnaire with six domains: physical functioning, role limitations, social functioning, pain, mental health, and vitality. The SF-6D score represents the strength of a patient’s preference for a particular health condition and ranges from zero (worst health state) to one (best health state).
Pittsburgh Sleep Quality Index (PSQI) [38]	Sleep quality	Questionnaire composed of 19 self-report questions and five questions that must be answered by a roommate or bed partner (which will not be used in this assessment). The 19 questions are classified into seven components (subjective sleep quality, sleep latency, sleep duration, habitual sleep efficiency, sleep disturbances, use of sleeping medication, and daytime dysfunction), which are classified into a score ranging from 0 (none in the last month) to 3 (three or more times/week). The sum of the scores for these seven components ranges from 0 to 21, where higher scores indicate poorer sleep quality. An overall score greater than 5 indicates great difficulty in at least two components, or moderate difficulty in more than three components.
Six-minute Walk Test (6MWT) [35–37]	Functional capacity	Before the test, the patient should rest for at least 10 min. The test will be performed on a flat, firm surface that is 30 m in length and seldom traveled. Two cones will be used to mark the starting point and the 30-m point where the participant must turn around and continue walking without hesitation. The surface will also have markings every 3 m with adhesive tape. The participants must walk for 6 min as fast as they can, without running, while the assessor times the walk (for 6 min) and counts how many laps are completed (each lap is 60 m). The participants will be allowed to slow down and/or stop during the test (including leaning against the wall) if necessary, but will be advised to walk again as soon as possible. Every minute of the test, the assessor will say the following words of encouragement: “You are doing well. You have x minutes to go”. With 15 s left to complete the test, the assessor will say: “In a moment I’m going to tell you to stop. When I do, just stop right where you are and I will come to you”. When the stopwatch is stopped, the assessor will say the word “stop”, go to the participant, and mark the point where he stopped with tape. After the test, the assessor will ask the patient to complete Borg scale. The distance traveled will be calculated by adding the number of laps to the meters walked until the participant stops.

### Economic assessment

Cost-effectiveness and cost-utility analysis will be carried out to evaluate the incremental cost per life-years adjusted for the quality (QALYs) of the interventions. The Fibromyalgia Impact Questionnaire will be used for cost-effectiveness analysis, and the EQ-5D-3L and SF-6D questionnaires will be used for cost-utility analysis. The costs measured in this study using resources by the participants will be: direct costs, which correspond to the costs of this participant to the public and private healthcare systems and the participant’s out-of-pocket expenses; and indirect costs through loss of productivity due to fibromyalgia. This assessment will be performed by information from a cost diary that the participants will keep at home. Three, six, nine, and 12 months after randomization, the assessor will collect these data by telephone. Thus, the costs of the interventions and the participants’ own expenses will be identified, measured, and validated. Table 2 shows the types of resources evaluated and the data sources from which the proposed information and evaluation methods will be taken. The number of work absences will be multiplied by the average salary rate to estimate the costs of paid work leave. Bus and subway fares or the price of fuel will be used to estimate transportation costs. Sensitivity analysis will test uncertainty in key parameters, such as the selection of cost weights and statistical variation in quality of life scores.

**Table 2:** Assessment of the resource used

Resource type	Assessment tool	Assessment method	Sources consulted
Physical therapy sessions	Physical therapist report	Wage rates	Physical therapy board fee schedule
Medications, visits to doctors and/or health professionals of the public and private health systems, hospital stay, visits to emergency medical services	Questionnaires every three months	Published prices (costs for public and private health systems) and/or actual costs to participants	Pharmaceutical websites and medical services fee schedule
Visits to community or alternative services or alternative health professionals	Questionnaires every three months	Actual costs to participants	Health services fee schedule
Loss of productivity (assessed for hours of lost work)	Questionnaires every three months	Average wage in the state of Sao Paulo per hour for women and men	Brazilian Institute of Geography and Statistics website
Transport, public or private, for the use of each resource	Questionnaires every three months	Values of bus and subway tickets and fuel in the year in the state of Sao Paulo.	Sptrans, metrosp, national agency for oil, natural gas and biofuels websites

### Randomization and concealed allocation

The randomization of the study will be performed by a researcher not involved in the recruitment of participants (CMNC), using Microsoft Excel for Windows. Allocation will be concealed and sealed in opaque envelopes in consecutively numbered sequences. Participants will be randomized to either the Aerobic Group or the Pilates Group.

### Interventions

Both groups will receive education through an educational booklet with information on fibromyalgia, central sensitization, self-care strategies for pain management and fatigue reduction (such as graduating activities of daily living and use of self-massage), improved sleep (such as avoiding caffeine and cigarette, and watching TV one hour before bedtime), improved depression and stress management (such as practicing physical activity and performing psychotherapy) [42, 43]. The booklet will also provide information about the importance of exercise in the management of fibromyalgia and delayed onset muscle soreness to reduce fear and avoidance of patients in relation to the practice of exercises. The participants may continue to use the medications prescribed by their doctor. The treatment will be carried out in person for eight weeks, with 16 individually tailored sessions lasting 60 min each. At each session, the participants will rate their pain before and after exercise. Any adverse effects caused by the exercises will be noted daily.

The Aerobic Group will perform aerobic exercises, and the training heart rate (HR) will be 57 to 76% of maximum heart rate (HRmax), which corresponds to the mild to moderate exercise intensity indicated for these patients [16, 25, 44]. HRmax will be calculated indirectly by the equation for maximum predicted heart rate by age (HRmax = 208–0.7 x age) [45, 46]. Patients should maintain training HR, which will be monitored by the frequencymeter, and a perceived effort between 11 and 13 (considered mild to moderate) [47] on the Borg scale [48]. The Borg scale will be the main factor considered when changing the intensity of the exercise if the patient presents a high perceived exertion and still does not reach the training HR. The indirect prescription of HRmax was chosen because it has been validated for sedentary patients with fibromyalgia and because it shows good correlation (*r* = 0.642; *p* < 0.05) with the HRmax obtained through ergospirometry [45]. The aerobic exercises will be done on a treadmill (model LX 3.0) or stationary bicycle (models LXU and LXR, Movement Fitness Equipment, Sao Paulo, SP, Brazil). The choice of treadmill or bicycle will be based on the comorbidities and preferences of the participant, because there are no differences between various aerobic exercise modalities in improving clinical outcomes in patients with fibromyalgia [14, 16, 17], and the chosen modality will be maintained until the end of the treatment [16]. The sessions will start with a warm-up (10-min walk) and will end with relaxation (10-min massage with a ball) with the patient lying down. The participants will be informed that they may have a tolerable increase in pain and fatigue in the short term, but these symptoms should return to baseline levels after the first weeks of exercise at adequate intensity [16]. The physical therapists responsible for aerobic exercise (EMESS and GCM) have two and seven years of undergraduate training, specialization in Sports Physical Therapy and Musculoskeletal Physical Therapy, and experience with the prescription of aerobic exercises.

The Pilates Group will receive a specific Pilates exercise program based on a previously published exercise booklet [49] with the inclusion of a few new exercises (Additional file 1). The sessions will include mat exercises with accessories as well as equipment-based exercises (Cadillac, Reformer, Ladder Barrel, and Step Chair, Metalife, Sao José, SC, Brazil). The last 10 min of the session will also consist of relaxation with a ball massage. In the first session, the participants will receive instructions on the method and will be trained on powerhouse activation through isometric contraction of the transverse abdominal, multifidus, and pelvic floor muscles associated with expiration [50, 51]. The exercises of the modified Pilates method will be executed in three levels of difficulty: basic, intermediate, and advanced. This level of difficulty will be defined individually and the participant’s progress between the levels will depend on how well they do the 10 repetitions of the exercises without postural compensations [52, 53]. The physical therapists responsible for Pilates exercises (KFMF and GCM) have six and ten years of undergraduate training, specialization in Musculoskeletal Physical Therapy, and seven and nine years of experience with the Pilates method.

The participants who are allocated to one group will not be transferred to the other group under any circumstances. If the participant is unable to perform the treatment after allocation, he will not do that specific exercise and will be reassessed according to the principles of intention-to-treat analysis. When the participants miss a session, the therapists will contact them by phone to schedule a make-up session. In these cases of absence, the 8-week protocol can be extended for up to 10 weeks to include the make-up sessions. All patients will receive refund for the money spent on public transportation to attend the treatment, which is a strategy to improve adherence in low income countries.

### Statistical analysis

All assessment forms will be scanned and stored in a safe place, and data tabulation will be saved on the study’s computer and on pen drives to avoid losses. The participants will be coded by numbers to maintain the confidentiality of their personal data, which will not be disclosed. One of the researchers (CMNC) will certify the data collection stage. The final results of the study will only be revealed upon completion of all follow-ups, and only the authors of the article will have access to the data prior to publication.

The data will be entered twice to avoid tabulation errors, and the statistician will receive the coded data and will be blind to group allocation. In both groups, descriptive analysis and evaluation of data normality will be performed through the visual inspection of histograms. The mean effects of the interventions and the differences between groups for all outcomes will be calculated using linear mixed models, which incorporate terms for the treatment groups, time (follow-ups), and interaction terms (treatment groups versus time). Time will be coded as a categorical variable (i.e., four variables will be created for the category baseline, eight weeks, six months, and 12 months). Treatment coefficients versus time interactions will be equivalent to the estimates of the differences between groups. The statistical analysis will use the software SPSS 19 for Windows.

The cost-effectiveness analysis will be performed using the impact of fibromyalgia as outcome, and the cost-utility analysis using QALYs. Mean between-group cost differences will be calculated for total and disaggregated costs. Seemingly unrelated regression analyses will be performed in which effect and cost differences will be corrected for their baseline values if available, while also taking into account the possible correlation between effects and costs [54]. Incremental cost-effectiveness and cost-utility ratios will be calculated by dividing the corrected difference in total costs by the difference in effects. Uncertainty surrounding the cost differences and incremental cost-effectiveness and cost-utility ratios will be estimated using Bias Corrected and Accelerated bootstrapping techniques (5000 replications). The latter will be graphically presented in cost-effectiveness planes [55]. Cost-effectiveness acceptability curves will be estimated to indicate the intervention’ probability of being cost-effective compared with other at different values of willingness-to-pay [56]. The economic evaluation will be performed using STATA V.14.

In addition, a survival analysis will be performed to evaluate the number of sessions necessary for a 30% pain reduction compared to the initial pain, and this value was chosen because it is considered a minimal clinically significant difference in pain in patients with fibromyalgia [57]. This analysis will be performed using the Kaplan-Meier method [58] and the comparison between the groups by the Log-Rank test [59]. All analyses will follow the intention-to-treat principle. The significance level will be set at 5%. Table 3 shows the timeline of the study.

**Table 3:** Timeline for the schedule of enrollment, interventions, and assessments

Outcomes	Enrollment	Before randomization	Intervention period (8 weeks)	8-week follow-up after randomization	6-month follow-up after randomization	12-month follow-up after randomization
Eligibility criteria	X					
Demographic data	X					
Informed consent	X					
Primary outcome						
Fibromyalgia impact		X		X		
Secondary outcomes						
Fibromyalgia impact					X	X
Pain intensity	X	X	X	X	X	X
Kinesiophobia		X		X	X	X
Specific disability		X		X	X	X
Health-related quality of life		X		X	X	X
Sleep quality		X		X	X	X
Functional capacity		X		X		
Interventions						
Aerobic exercise			X			
Pilates exercise			X			

## Discussion

Until the moment, this is the most robust and least biased study about the effectiveness of the Pilates method as a treatment resource for patients with fibromyalgia, comparing it with aerobic exercise. It is also the first study to investigate the cost-effectiveness of two different modalities of therapeutic exercise. Current evidence shows that aerobic exercise does not generate very good adherence in the treatment of patients with fibromyalgia [60], although it has a low to moderate effect on the improvement of their symptoms [16]. Pilates method can lead to greater adherence [61] because it uses equipment and exercises divided into levels of progression. Pilates exercises may cause less muscle soreness and may be more pleasurable and stimulating, which would facilitate its implementation in patients with fibromyalgia who tend to be resistant to exercise. Finally, there is little information on the long-term effects of different exercise modalities for patients with fibromyalgia, and this study will monitor these patients for one year.

This study aims to clarify whether the Pilates method can be incorporated into the clinical practice of physical therapists treating patients with fibromyalgia, as well as which exercises to use and how these exercises can be performed. An easy-to-replicate protocol for aerobic exercise in these patients, with adequate training intensity and frequency, will also be described, as well as information about muscle soreness pain and progression of exercises in each exercise modality. The study will also provide information on which exercise will be most cost-effective, information that can be used by insurers and public health systems. With this study, patients will know whether the Pilates method can be used as an alternative treatment and which exercise modality causes less pain.

The study took all measures to minimize the risks of methodological bias, that is, it was prospectively registered, and it has a relevant sample, concealed allocation, intention-to-treat analysis, and blinded assessor. It allows generalization of the data for the majority of patients with fibromyalgia. In addition, it has pragmatic interventions, with Pilates exercises being performed as in clinical practice, with individualized exercises according to the clinical conditions of the participants each day, without a fixed protocol. The aerobic exercise will be prescribed following the recommendations of the most recent systematic reviews [16, 25] and the American College of Sports Medicine [44], respecting the individuality and clinical condition of each participant by monitoring exercise intensity. The physical therapists responsible for the interventions have clinical experience in the prescription of the respective exercises. The study has as a limitation the non-blinding of participants and therapists, and this limitation stems from the type of intervention (physical exercise).

## Additional file

Additional file 1: Exercises added and altered from the previously published exercise booklet^49^. (DOCX 1702 kb)

## Abbreviations

EQ-5D-3L: Euroqol 5 dimensions
HR: Heart rate
HRmax: Maximum heart rate
QALY: Quality adjusted life years
SF-6D: Short-form 6 dimensions
SS: Severity of symptoms
WPI: Widespread pain index
