# Supplementary material for: Effectiveness and cost-effectiveness of the modified Pilates method versus aerobic exercise in the treatment of patients with fibromyalgia: protocol for a randomized controlled trial
Source: BMC Rheumatol. 2019 Jan 18;3:2. doi: 10.1186/s41927-018-0051-6 (PMC6390629; doi:10.1186/s41927-018-0051-6)
Supplement: Supplementary file 1 — Exercises added and altered from the previously published exercise booklet49. (DOCX 1702 kb) [file 41927_2018_51_MOESM1_ESM.docx]

**Additional file 1** – Exercises added and altered from the previously published exercise booklet^49^

| **Warm-up Exercises (Before Pilates)** | | | |
| --- | --- | --- | --- |
| *Breathing*  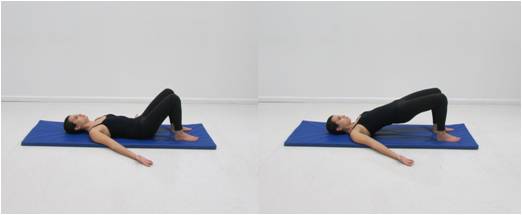  Objective: to teach and remind patients of the three-dimensional breathing and the principle of Pilates breathing. | *Pelvic mobility*    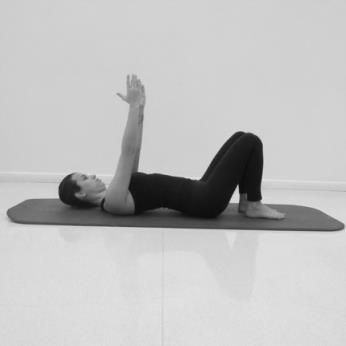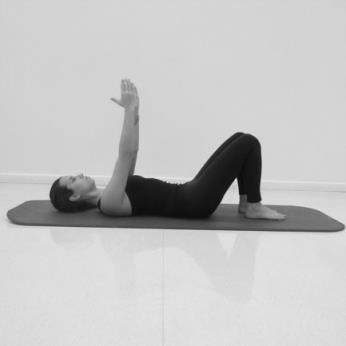  Objective: to teach the neutral position of the lumbar spine and the principle of centralization, mobilize pelvis and lumbar spine and strengthen the gluteal and hamstring muscles. | | *Hip mobility*    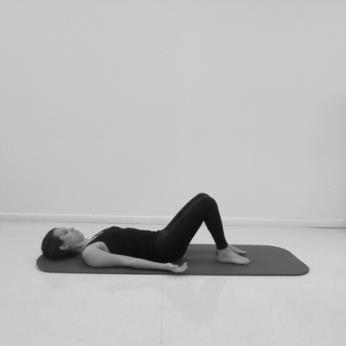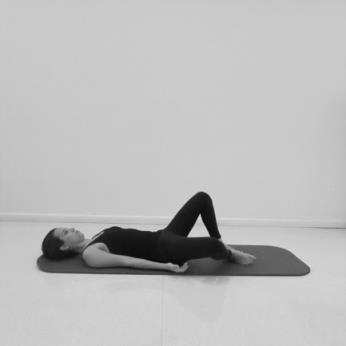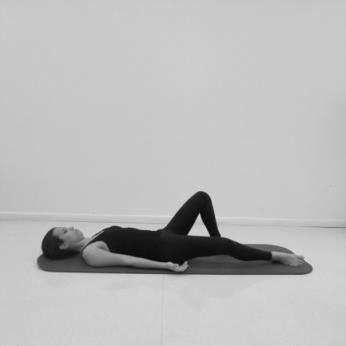  Objective: to control the pelvis in neutral position and to mobilize the hip. |
| *Shoulder and scapular mobility*  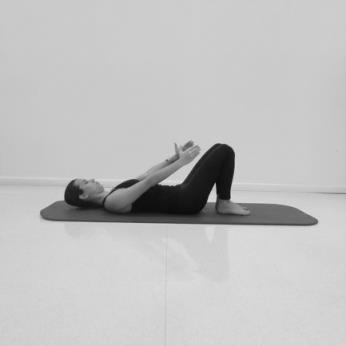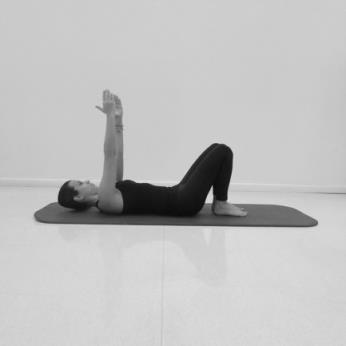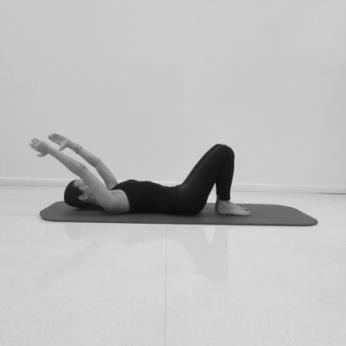  Objective: to mobilize the shoulder, teach scapular and cervical awareness, teach three-dimensional breathing and centralization to prevent the opening of the ribs during the raising of the arms. | | *Abdomen preparation*  **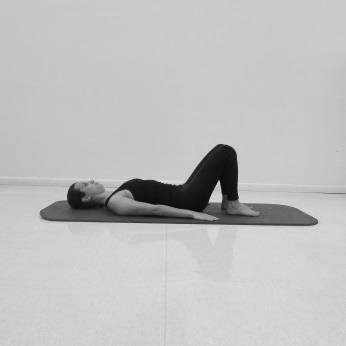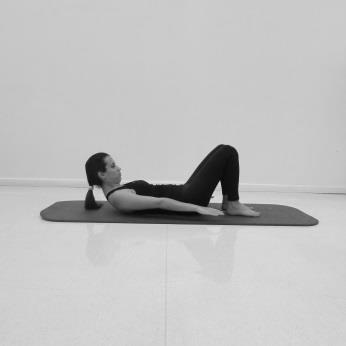**  Objective: to teach cervical awareness with the elevation of the trunk, strengthen the rectus abdominis, internal oblique and external oblique muscles. | |

| **Exercises for the Back** | | |
| --- | --- | --- |
| **Basic** | **Intermediary** | **Advanced** |
| *Bridge Variant*  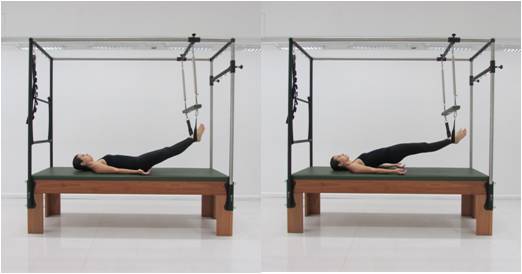  Objective: to mobilize the pelvis and spine and strengthen the hamstrings and gluteus muscles. | *Bridge Variant*  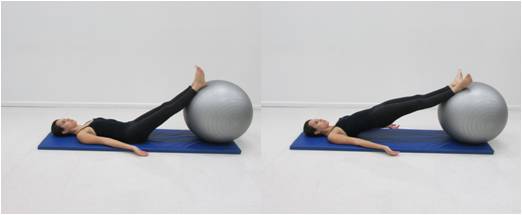  Objective: to mobilize the pelvis and spine and strengthen the gluteus, hamstrings and gastrocnemius muscles. | 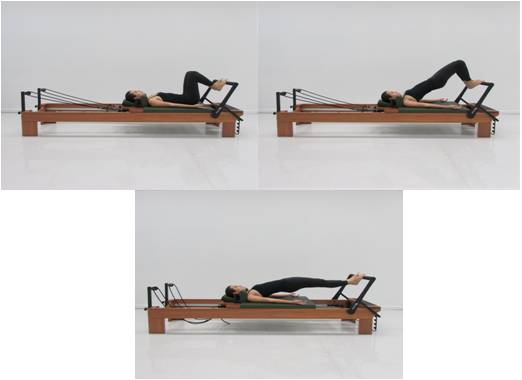*Bridge Variant*  Objective: to strengthen the hamstrings, quadriceps femoral, calf, iliopsoas, sartorius, tensor fascia latae, pectineus and gluteus muscles. |
| *Bridge*  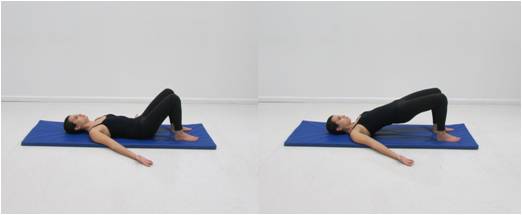  Objective: to mobilize the pelvis and spine and strengthen the gluteus and hamstrings muscles. | *Bridge* *Variant with balance disk* 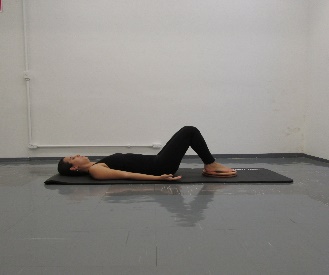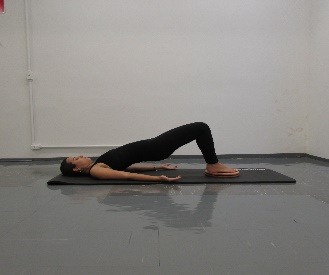 Objective: to mobilize the pelvis and spine and strengthen the gluteus and hamstrings muscles. | *Bridge* *Variant*  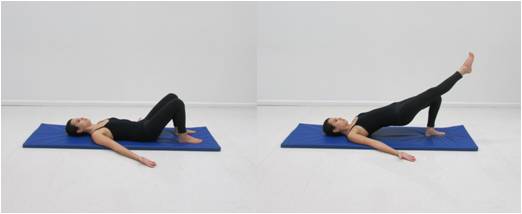  Objective: to mobilize the pelvis and spine and strengthen the hamstrings, gluteus and quadriceps femoris muscles. |

| *Rolling Back*  *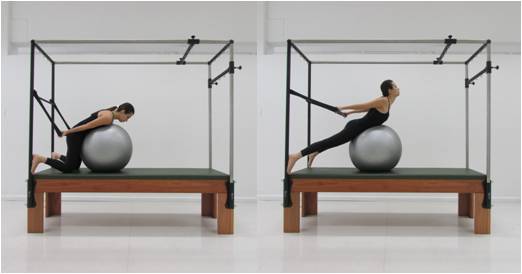*  Objective: to mobilize the spine, stretch the muscles from the anterior-internal chain of the shoulder and activate the abdominal muscles associated with the powerhouse. | *Rolling Back Variant*  *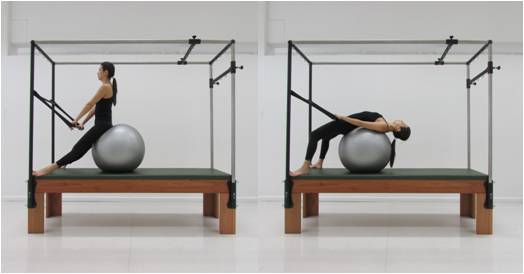*  Objective: to mobilize the spine and strengthen the rectus abdominis, internal oblique and external oblique muscles associated with the powerhouse. | *Rolling Back Variant* 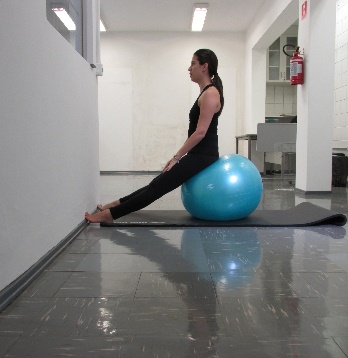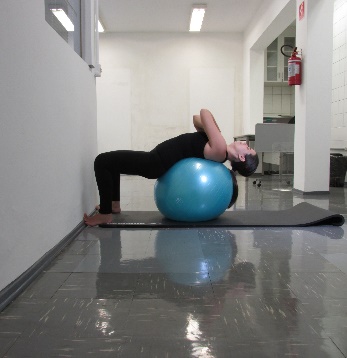 Objective: to mobilize the spine and strengthen the rectus abdominis, internal oblique and external oblique muscles associated with the powerhouse. |
| --- | --- | --- |
| *Low back twist*  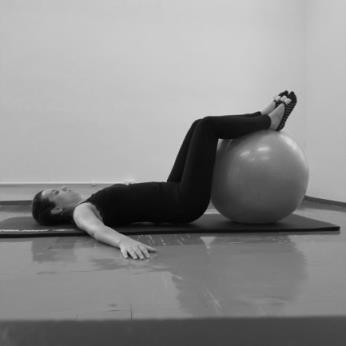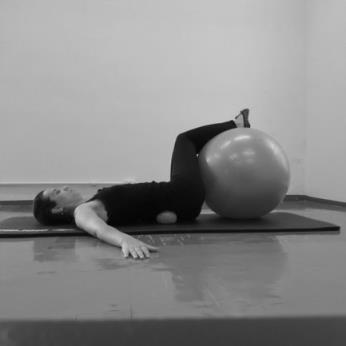  Objective: to relax lumbar muscles and strengthen abdominal muscles. | *Low back twist* 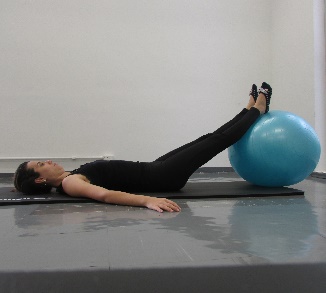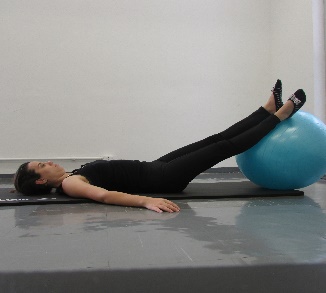 Objective: to relax lumbar muscles and strengthen abdominal muscles. | *Low back twist*  **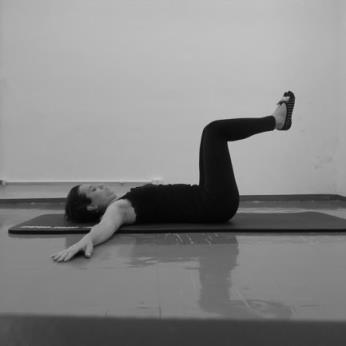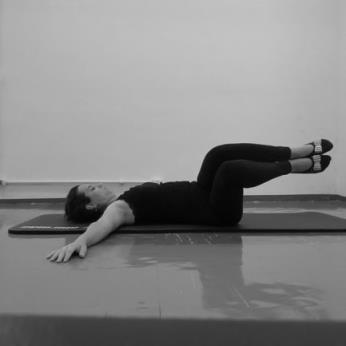**  Objective: to relax lumbar muscles and strengthen abdominal muscles. |
| *Head bending and extension in supine*  **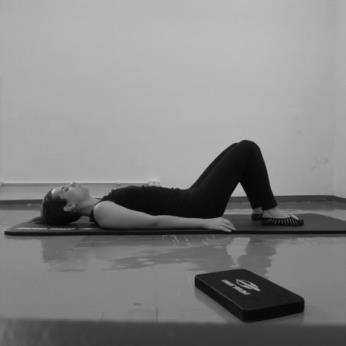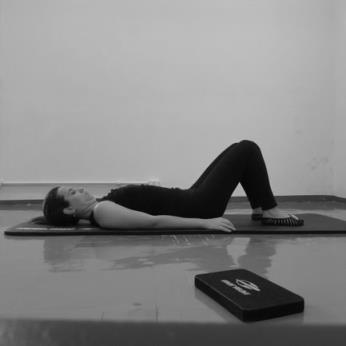**  Objective: to conscientize the correct movement of the cervical spine, to mobilize cervical spine and activate the stabilizing muscles of the cervical spine. | *Head bending and extension in ventral*  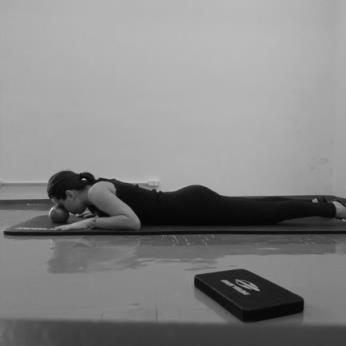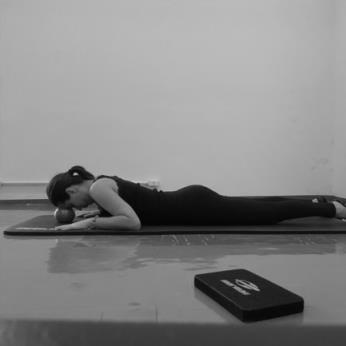  Objective: to conscientize the correct movement of the cervical spine, to mobilize cervical spine and activate the stabilizing muscles of the cervical spine. | *Neck retraction*  *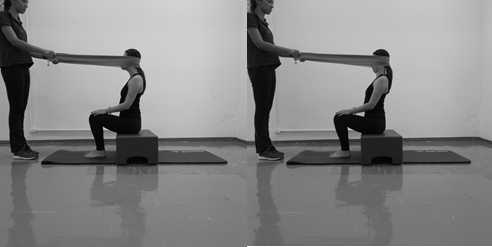*  Objective: to conscientize the correct movement of the cervical spine, to mobilize cervical spine and strengthen the stabilizing muscles of the cervical spine. |
| *Thoracic mobility*  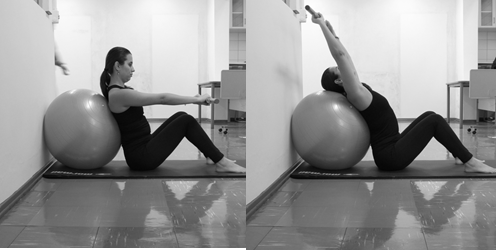  Objective: to mobilize cervical and thoracic spine, stretch the muscles from the anterior chain of the shoulder and relaxation of thoracic spine extensor muscles. | *Thoracic mobility* 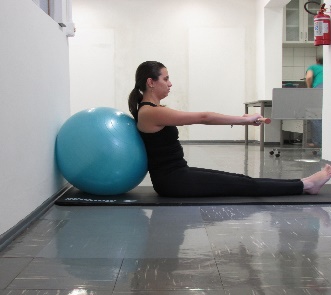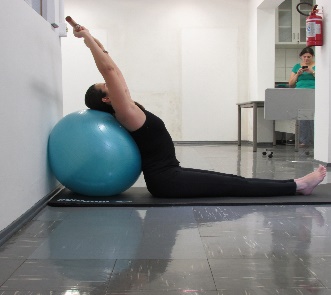 Objective: to mobilize cervical and thoracic spine, stretch the muscles from the anterior chain of the shoulder and relaxation of thoracic spine extensor muscles. | *Thoracic mobility*  **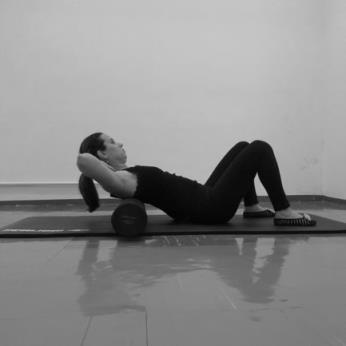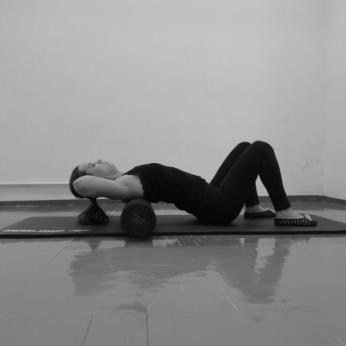**  Objective: to mobilize cervical and thoracic spine, stretch the muscles from the anterior chain of the shoulder and strengthen the abdominal muscles. |

| *Trapezius stretching*  *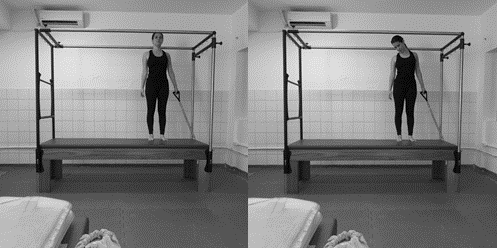*  Objective: to stretch the ipsilateral upper trapezius muscle and provide muscle relaxation. | *Trapezius stretching* 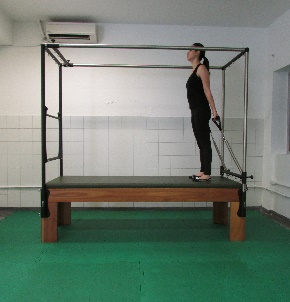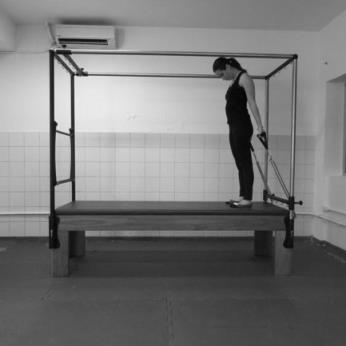 Objective: to stretch the ipsilateral upper trapezius muscle and provide muscle relaxation. | *Trapezius stretching*  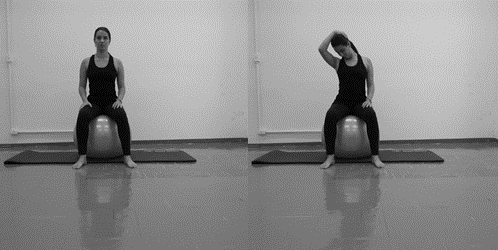  Objective: to stretch the ipsilateral upper trapezius muscle. and train balance. |
| --- | --- | --- |

| **Exercises for the Lower Limbs** | | | | |
| --- | --- | --- | --- | --- |
| **Basic** | | **Intermediary** | | **Advanced** |
| *Tower*  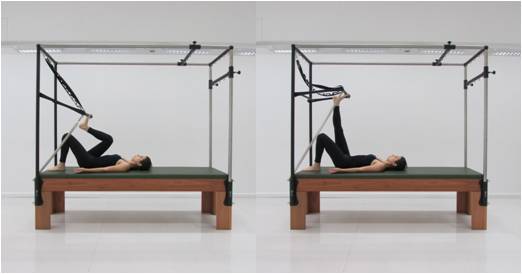  Objective: to stretch the muscles from the posterior chain and strengthen the quadriceps femoris and gluteus maximus muscles. | | *Tower*    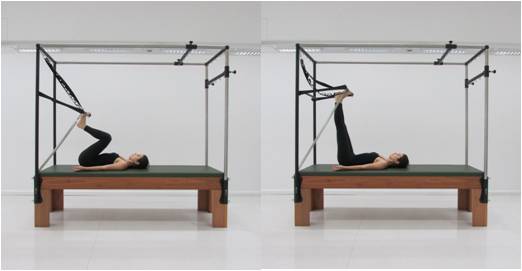  Objective: to stretch the muscles from the posterior chain and strengthen the quadriceps femoris and gluteus maximus muscles. | | *Tower*    *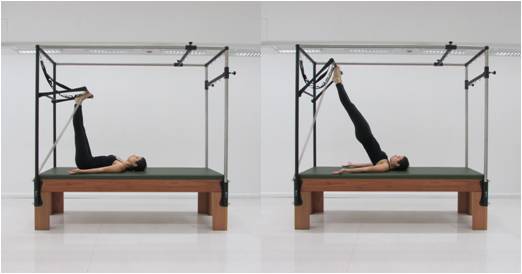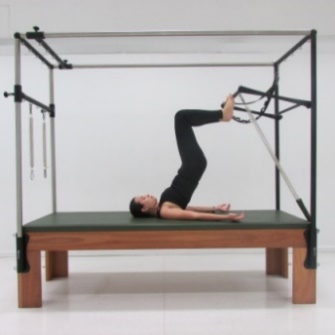*  Objective: to mobilize spine, strengthen the gluteus maximus, hamstrings and triceps surae, rectus abdominis, internal and external oblique muscles. |
| *Leg Circles with half moon*  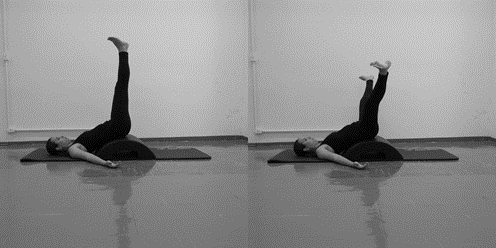  Objective: to strengthen the quadriceps femoris, iliopsoas, sartorius, tensor fascia latae, pectineus, gluteus medius and minimus, adductor magnus and longus, gracilis muscles. | | *Leg Series Supine Circle*  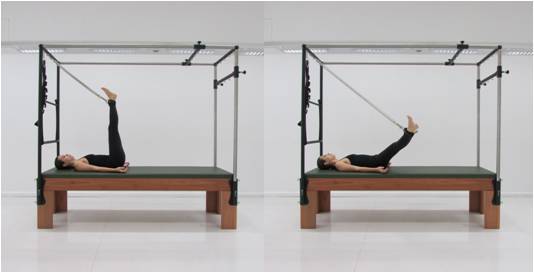  Objective: to strengthen the gluteus maximus, hamstrings, adductor magnus and longus, pectineus and gracilis muscles. | | *Leg Circles*    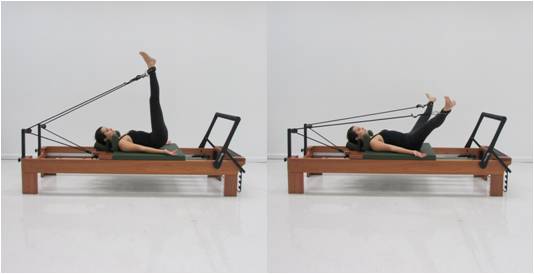  Objective: to strengthen the gluteus maximus, hamstrings, adductor magnus and longus, pectineus and gracilis muscles. |
| **Exercises for the Upper Limbs** | | | | |
| **Basic** | **Intermediary** | | **Advanced** | |
| *Arms Biceps*  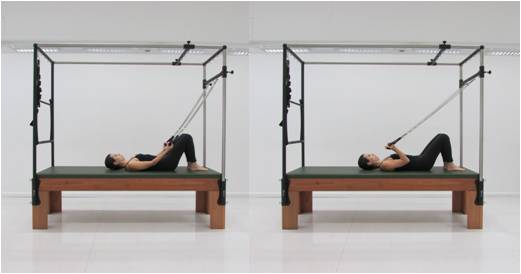  Objective: to strengthen the biceps brachii and brachialis muscles. | *Arms Biceps Variant*  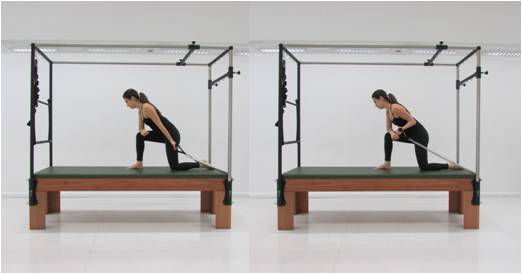  Objective: to strengthen the biceps brachii, brachialis, rectus abdominal, iliopsoas and rectus femoris muscles. | | *Arms Biceps Variant*  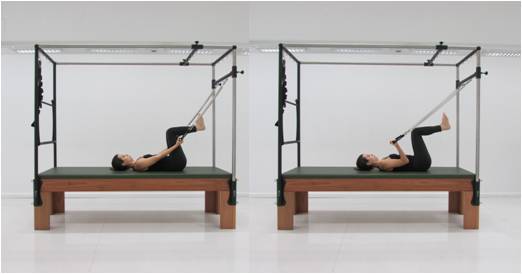  Objective: to strengthen the biceps brachii, brachialis, rectus abdominis, internal and external oblique muscles. | |
| *Arms Triceps*  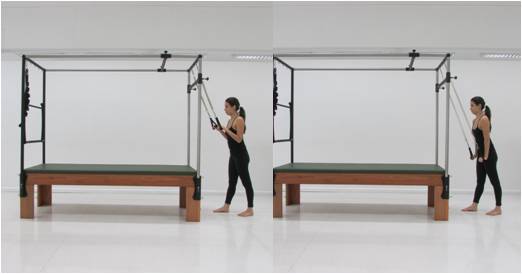  Objective: to strengthen the triceps brachii and anconeus muscles. | *Arm Triceps*  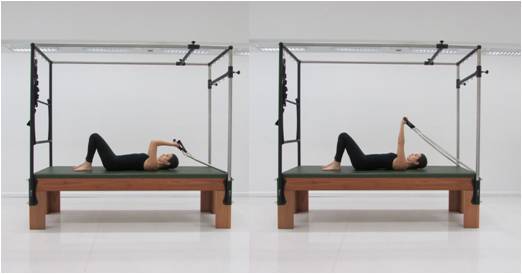  Objective: to strengthen the triceps brachii and anconeus muscles. | | *Standing On Floor At Open End Boxe*    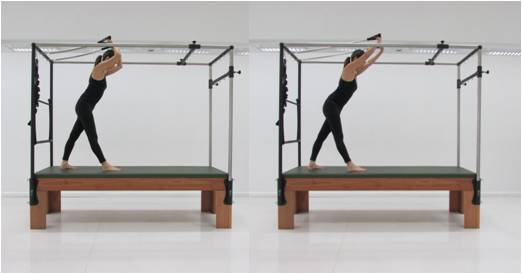  Objective: to strengthen the triceps brachii and anconeus muscles. | |
| *Pectoralis stretching*  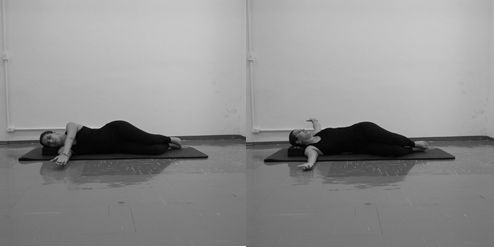  Objective: to stretch the pectoralis major and minor, coracobrachialis and anterior portion of the deltoid. | *Pectoralis stretching*  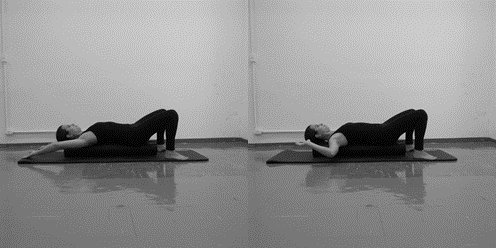  Objective: to stretch the pectoralis major and minor, coracobrachialis and anterior portion of the deltoid. | | *Pectoralis stretching*  *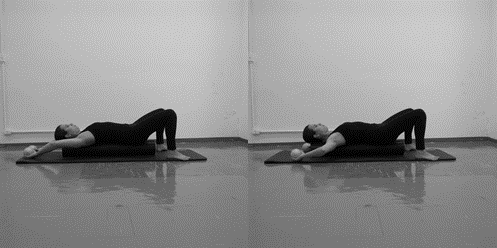*  Objective: to stretch the pectoralis major and minor, coracobrachialis and anterior portion of the deltoid. | |
